# 1-(5-Bromo-2-oxoindolin-3-yl­idene)thio­semicarbazide acetonitrile monosolvate

**DOI:** 10.1107/S1600536811023786

**Published:** 2011-06-25

**Authors:** Fernanda Rosi Soares Pederzolli, Leandro Bresolin, Vanessa Santana Carratu, Aline Locatelli, Adriano Bof de Oliveira

**Affiliations:** a Escola de Química e Alimentos, Universidade Federal do Rio Grande, Av. Itália km 08, Campus Carreiros, 96201-900 Rio Grande, RS, Brazil; b Departamento de Química, Universidade Federal de Santa Maria, Av. Roraima, Campus, 97105-900 Santa Maria, RS, Brazil; c Departamento de Química, Universidade Federal de Sergipe, Av. Marechal Rondon s/n, Campus, 49100-000 São Cristóvão-SE, Brazil

## Abstract

In the crystal structure of the title compound, C_9_H_7_BrN_4_OS·C_2_H_3_N, the mol­ecules are connected *via* N—H⋯O and N—H⋯S inter­actions into zigzag chains perpendicular to [001]. The mol­ecules in these chains are additionally linked to acetonitrile solvent mol­ecules through N—H⋯N hydrogen bonding. The mol­ecules are arranged in layers and are stacked in the direction of the *c* axis indicative of π–π inter­actions, with distance = 3.381 (7) Å for the C⋯C interaction parallel to [001]. An intra­molecular N—H⋯O hydrogen bond is also observed in the main mol­ecule.

## Related literature

For the pharmacological properties of isatin-thio­semicarbazone derivatives against cruzain, falcipain-2 and rhodesain, see: Chiyanzu *et al.* (2003 ▶). For the synthesis of 5-bromo­isatin-3-thio­semicarbazone, see: Campaigne & Archer (1952 ▶).
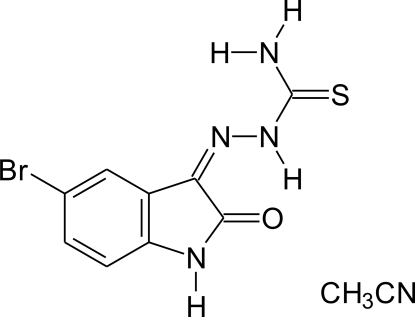


## Experimental

### 

#### Crystal data

C_9_H_7_BrN_4_OS·C_2_H_3_N
*M* *_r_* = 340.21
Monoclinic, 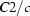

*a* = 20.017 (4) Å
*b* = 13.352 (2) Å
*c* = 13.190 (5) Å
β = 129.258 (2)°
*V* = 2729.6 (12) Å^3^
*Z* = 8
Mo *K*α radiation
μ = 3.16 mm^−1^
*T* = 293 K
0.22 × 0.20 × 0.16 mm

#### Data collection

Bruker CCD X8 APEXII diffractometer
10884 measured reflections
3377 independent reflections
2754 reflections with *I* > 2σ(*I*)
*R*_int_ = 0.030

#### Refinement

*R*[*F* ^2^ > 2σ(*F* ^2^)] = 0.039
*wR*(*F* ^2^) = 0.105
*S* = 1.09
3377 reflections
173 parameters
H-atom parameters constrained
Δρ_max_ = 1.03 e Å^−3^
Δρ_min_ = −0.86 e Å^−3^

### 

Data collection: *APEX2* (Bruker, 2006 ▶); cell refinement: *SAINT* (Bruker, 2003 ▶); data reduction: *SAINT*; program(s) used to solve structure: *SHELXS97* (Sheldrick, 2008 ▶); program(s) used to refine structure: *SHELXL97* (Sheldrick, 2008 ▶); molecular graphics: *DIAMOND* (Brandenburg, 2006 ▶); software used to prepare material for publication: *publCIF* (Westrip, 2010 ▶).

## Supplementary Material

Crystal structure: contains datablock(s) I, global. DOI: 10.1107/S1600536811023786/nc2233sup1.cif

Structure factors: contains datablock(s) I. DOI: 10.1107/S1600536811023786/nc2233Isup2.hkl

Supplementary material file. DOI: 10.1107/S1600536811023786/nc2233Isup3.cml

Additional supplementary materials:  crystallographic information; 3D view; checkCIF report

## Figures and Tables

**Table 1:** Hydrogen-bond geometry (Å, °)

*D*—H⋯*A*	*D*—H	H⋯*A*	*D*⋯*A*	*D*—H⋯*A*
N3—H5⋯O	0.86	2.10	2.769 (3)	134
N4—H6⋯N5	0.86	2.61	3.438 (5)	161
N4—H7⋯O^i^	0.86	2.05	2.906 (4)	173
N1—H4⋯S^ii^	0.86	2.50	3.350 (3)	169

Symmetry codes: (i) 
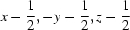
; (ii) 
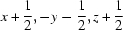
.

**Fig. 1.**
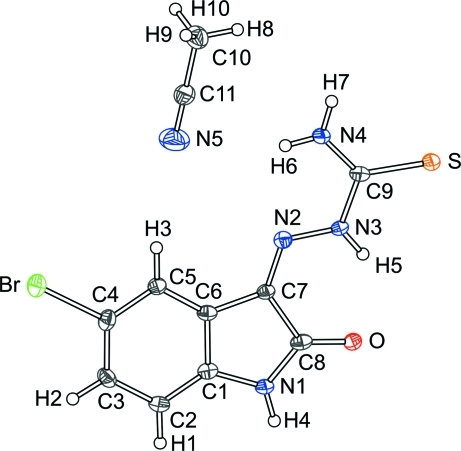
: The molecular structure of the title compound with labelling and displacement ellipsoids drawn at the 50% probability level.

**Fig. 2.**
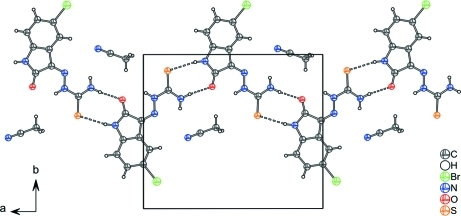
: Crystal structure of the title compound viewed in the direction of the crystallographic c axis. Hydrogen bonding is indicated as dashed lines.

## supplementary crystallographic information

### Comment

Thiosemicarbazone derivatives have a wide range of biological properties. For example, isatin-based synthetic thiosemicarbazones show pharmacological activity against cruzain, falcipain-2 and rhodesain (Chiyanzu *et al.*, 2003). As part of our study of thiosemicarbazone derivatives, we report herein the crystal structure of 5-Bromoisatin-3-thiosemicarbazone acetonitrile solvate.

The crystal structure of the title compound is build of one-dimensional zigzag chain in which the molecules are linked by pairs of N—H···O and N—H···S hydrogen bonding. Each two molecules within these chains are additionally linked by acetonitrile molecules *via* N—H···N hydrogen bonding and weak C—H···S interactions. The molecules are arranged in layers and are stacked into the direction of the *c*-axis indicative for π-π-interactions.

### Experimental

Starting materials were commercially available and were used without further purification. The synthesis was adapted from a procedure reported previously (Campaigne & Archer, 1952). The hydrochloric acid catalyzed reaction of 5-bromoisatin (8,83 mmol) and thiosemicarbazide (8,83 mmol) in ethanol (50 ml) was refluxed for 6 h. After cooling and filtering, crystals suitable for X-ray diffraction were obtained from an acetonitrile solution.

### Refinement

The C-H and N-H H atoms were positioned with idealized geometry and were refined isotropic with  *U*_eq_(H) set to 1.2 times of the *U*_eq_ of the parent atom (1.5 for methyl H atoms) using a riding model with C—H = 0.93 Å for aromatic), C—H = 0.96 Å for methyl and N—H = 0.86 Å for N-H H atoms.

### Crystal data

**Table:**

C_9_H_7_BrN_4_OS·C_2_H_3_N	*F*(000) = 1360
*M**_r_* = 340.21	*D*_x_ = 1.656 Mg m^−^^3^
Monoclinic, *C*2/*c*	Melting point: 544.15 K
Hall symbol: -C 2yc	Mo *K*α radiation, λ = 0.71073 Å
*a* = 20.017 (4) Å	Cell parameters from 3718 reflections
*b* = 13.352 (2) Å	θ = 2.6–27.6°
*c* = 13.190 (5) Å	µ = 3.16 mm^−^^1^
β = 129.258 (2)°	*T* = 293 K
*V* = 2729.6 (12) Å^3^	Block, yellow
*Z* = 8	0.22 × 0.20 × 0.16 mm

### Data collection

**Table:**

Bruker CCD X8 APEXII diffractometer	2754 reflections with *I* > 2σ(*I*)
Radiation source: fine-focus sealed tube, Bruker CCD X8 APEXII	*R*_int_ = 0.030
graphite	θ_max_ = 28.3°, θ_min_ = 2.0°
φ and ω scans	*h* = −26→14
10884 measured reflections	*k* = −16→17
3377 independent reflections	*l* = −7→17

### Refinement

**Table:**

Refinement on *F*^2^	Primary atom site location: structure-invariant direct methods
Least-squares matrix: full	Secondary atom site location: difference Fourier map
*R*[*F*^2^ > 2σ(*F*^2^)] = 0.039	Hydrogen site location: inferred from neighbouring sites
*wR*(*F*^2^) = 0.105	H-atom parameters constrained
*S* = 1.09	*w* = 1/[σ^2^(*F*_o_^2^) + (0.0396*P*)^2^ + 12.0809*P*] where *P* = (*F*_o_^2^ + 2*F*_c_^2^)/3
3377 reflections	(Δ/σ)_max_ < 0.001
173 parameters	Δρ_max_ = 1.03 e Å^−^^3^
0 restraints	Δρ_min_ = −0.86 e Å^−^^3^

### Special details

**Table:**

Geometry. All e.s.d.'s (except the e.s.d. in the dihedral angle between two l.s. planes) are estimated using the full covariance matrix. The cell e.s.d.'s are taken into account individually in the estimation of e.s.d.'s in distances, angles and torsion angles; correlations between e.s.d.'s in cell parameters are only used when they are defined by crystal symmetry. An approximate (isotropic) treatment of cell e.s.d.'s is used for estimating e.s.d.'s involving l.s. planes.
Refinement. Refinement of *F*^2^ against ALL reflections. The weighted *R*-factor *wR* and goodness of fit *S* are based on *F*^2^, conventional *R*-factors *R* are based on *F*, with *F* set to zero for negative *F*^2^. The threshold expression of *F*^2^ > σ(*F*^2^) is used only for calculating *R*-factors(gt) *etc*. and is not relevant to the choice of reflections for refinement. *R*-factors based on *F*^2^ are statistically about twice as large as those based on *F*, and *R*- factors based on ALL data will be even larger.

### Fractional atomic coordinates and isotropic or equivalent isotropic displacement parameters (Å^2^)

**Table:**

	*x*	*y*	*z*	*U*_iso_*/*U*_eq_	
C9	0.3611 (2)	−0.2695 (2)	0.4897 (3)	0.0175 (6)	
C10	0.0960 (2)	−0.0277 (3)	0.2196 (3)	0.0297 (8)	
H8	0.0967	−0.0995	0.2159	0.045*	
H9	0.0735	−0.0003	0.1361	0.045*	
H10	0.0602	−0.0075	0.2410	0.045*	
C11	0.1832 (2)	0.0088 (3)	0.3192 (4)	0.0296 (8)	
C6	0.52345 (19)	0.0354 (2)	0.6478 (3)	0.0166 (6)	
C5	0.4655 (2)	0.1144 (2)	0.5889 (3)	0.0169 (6)	
H3	0.4062	0.1038	0.5301	0.020*	
C4	0.5007 (2)	0.2109 (2)	0.6225 (3)	0.0205 (6)	
C3	0.5890 (2)	0.2282 (2)	0.7097 (3)	0.0214 (7)	
H2	0.6101	0.2934	0.7300	0.026*	
C2	0.6469 (2)	0.1459 (3)	0.7675 (3)	0.0224 (7)	
H1	0.7063	0.1558	0.8254	0.027*	
C1	0.6126 (2)	0.0514 (2)	0.7353 (3)	0.0181 (6)	
C8	0.5995 (2)	−0.1188 (3)	0.7243 (3)	0.0191 (6)	
C7	0.51065 (19)	−0.0725 (2)	0.6367 (3)	0.0152 (6)	
Br	0.42473 (2)	0.32205 (3)	0.54837 (4)	0.02739 (12)	
N2	0.43754 (17)	−0.1178 (2)	0.5672 (2)	0.0171 (5)	
N3	0.43806 (16)	−0.2191 (2)	0.5678 (2)	0.0174 (5)	
H5	0.4860	−0.2515	0.6165	0.021*	
N4	0.29129 (18)	−0.2149 (2)	0.4244 (3)	0.0217 (6)	
H6	0.2951	−0.1507	0.4317	0.026*	
H7	0.2414	−0.2430	0.3741	0.026*	
N5	0.2515 (2)	0.0381 (3)	0.3981 (4)	0.0491 (10)	
N1	0.65560 (17)	−0.0418 (2)	0.7786 (3)	0.0197 (5)	
H4	0.7108	−0.0486	0.8329	0.024*	
O	0.61642 (14)	−0.20772 (17)	0.7432 (2)	0.0200 (5)	
S	0.36470 (5)	−0.39515 (6)	0.48661 (8)	0.02291 (19)	

### Atomic displacement parameters (Å^2^)

**Table:**

	*U*^11^	*U*^22^	*U*^33^	*U*^12^	*U*^13^	*U*^23^
C9	0.0139 (15)	0.0224 (17)	0.0165 (14)	−0.0033 (12)	0.0098 (13)	−0.0014 (12)
C10	0.0273 (19)	0.0265 (19)	0.0264 (18)	−0.0018 (15)	0.0126 (16)	−0.0003 (15)
C11	0.0214 (18)	0.0231 (18)	0.035 (2)	0.0042 (14)	0.0134 (17)	0.0086 (15)
C6	0.0129 (14)	0.0219 (16)	0.0136 (14)	−0.0017 (12)	0.0077 (12)	−0.0009 (12)
C5	0.0157 (15)	0.0187 (15)	0.0156 (14)	0.0004 (12)	0.0097 (13)	0.0004 (12)
C4	0.0239 (17)	0.0175 (16)	0.0227 (16)	0.0048 (13)	0.0160 (15)	0.0041 (13)
C3	0.0248 (17)	0.0163 (16)	0.0259 (17)	−0.0049 (13)	0.0173 (15)	−0.0006 (13)
C2	0.0138 (15)	0.0238 (17)	0.0244 (17)	−0.0037 (13)	0.0096 (14)	−0.0032 (13)
C1	0.0149 (15)	0.0230 (16)	0.0164 (14)	−0.0006 (12)	0.0099 (13)	−0.0005 (12)
C8	0.0124 (15)	0.0271 (18)	0.0147 (14)	−0.0001 (13)	0.0072 (13)	0.0000 (12)
C7	0.0119 (14)	0.0188 (15)	0.0128 (13)	0.0001 (11)	0.0068 (12)	−0.0005 (11)
Br	0.0271 (2)	0.01948 (18)	0.0320 (2)	0.00440 (14)	0.01693 (16)	0.00453 (14)
N2	0.0164 (13)	0.0179 (13)	0.0172 (13)	0.0003 (10)	0.0107 (12)	0.0007 (10)
N3	0.0101 (12)	0.0179 (13)	0.0187 (13)	0.0011 (10)	0.0066 (11)	0.0003 (10)
N4	0.0130 (13)	0.0167 (13)	0.0283 (15)	−0.0002 (11)	0.0097 (12)	0.0017 (11)
N5	0.0247 (19)	0.046 (2)	0.052 (2)	−0.0026 (16)	0.0130 (18)	0.0101 (19)
N1	0.0110 (12)	0.0191 (14)	0.0223 (13)	−0.0005 (10)	0.0073 (11)	−0.0002 (11)
O	0.0133 (11)	0.0176 (11)	0.0212 (11)	0.0014 (9)	0.0072 (10)	0.0005 (9)
S	0.0140 (4)	0.0176 (4)	0.0273 (4)	0.0001 (3)	0.0084 (3)	0.0001 (3)

### Geometric parameters (Å, °)

**Table:**

C9—N4	1.305 (4)	C3—C2	1.419 (5)
C9—N3	1.370 (4)	C3—H2	0.9300
C9—S	1.681 (3)	C2—C1	1.369 (5)
C10—C11	1.451 (5)	C2—H1	0.9300
C10—H8	0.9600	C1—N1	1.412 (4)
C10—H9	0.9600	C8—O	1.217 (4)
C10—H10	0.9600	C8—O	1.217 (4)
C11—N5	1.142 (5)	C8—N1	1.346 (4)
C11—N5	1.142 (5)	C8—C7	1.510 (4)
C6—C5	1.386 (4)	C7—N2	1.285 (4)
C6—C1	1.398 (4)	N2—N3	1.352 (4)
C6—C7	1.455 (4)	N3—H5	0.8600
C5—C4	1.399 (5)	N4—H6	0.8600
C5—H3	0.9300	N4—H7	0.8600
C4—C3	1.389 (5)	N1—H4	0.8600
C4—Br	1.894 (3)		
			
N4—C9—N3	116.5 (3)	C1—C2—H1	121.0
N4—C9—S	125.9 (2)	C3—C2—H1	121.0
N3—C9—S	117.6 (2)	C2—C1—C6	121.6 (3)
C11—C10—H8	109.5	C2—C1—N1	129.0 (3)
C11—C10—H9	109.5	C6—C1—N1	109.4 (3)
H8—C10—H9	109.5	O—C8—O	0.00 (5)
C11—C10—H10	109.5	O—C8—N1	127.3 (3)
H8—C10—H10	109.5	O—C8—N1	127.3 (3)
H9—C10—H10	109.5	O—C8—C7	126.6 (3)
N5—C11—N5	0.0 (8)	O—C8—C7	126.6 (3)
N5—C11—C10	179.2 (5)	N1—C8—C7	106.1 (3)
N5—C11—C10	179.2 (5)	N2—C7—C6	125.9 (3)
C5—C6—C1	121.7 (3)	N2—C7—C8	127.7 (3)
C5—C6—C7	131.7 (3)	C6—C7—C8	106.3 (3)
C1—C6—C7	106.6 (3)	C7—N2—N3	117.8 (3)
C6—C5—C4	116.6 (3)	N2—N3—C9	119.0 (3)
C6—C5—H3	121.7	N2—N3—H5	120.5
C4—C5—H3	121.7	C9—N3—H5	120.5
C3—C4—C5	122.5 (3)	C9—N4—H6	120.0
C3—C4—Br	118.8 (3)	C9—N4—H7	120.0
C5—C4—Br	118.6 (2)	H6—N4—H7	120.0
C4—C3—C2	119.6 (3)	C8—N1—C1	111.6 (3)
C4—C3—H2	120.2	C8—N1—H4	124.2
C2—C3—H2	120.2	C1—N1—H4	124.2
C1—C2—C3	117.9 (3)		
			
C1—C6—C5—C4	−0.6 (4)	O—C8—C7—N2	0.2 (5)
C7—C6—C5—C4	−180.0 (3)	N1—C8—C7—N2	−178.7 (3)
C6—C5—C4—C3	0.3 (4)	O—C8—C7—C6	179.1 (3)
C6—C5—C4—Br	−178.6 (2)	O—C8—C7—C6	179.1 (3)
C5—C4—C3—C2	0.3 (5)	N1—C8—C7—C6	0.2 (3)
Br—C4—C3—C2	179.2 (2)	C6—C7—N2—N3	179.4 (3)
C4—C3—C2—C1	−0.7 (5)	C8—C7—N2—N3	−1.9 (4)
C3—C2—C1—C6	0.4 (5)	C7—N2—N3—C9	−176.9 (3)
C3—C2—C1—N1	179.9 (3)	N4—C9—N3—N2	−3.6 (4)
C5—C6—C1—C2	0.3 (5)	S—C9—N3—N2	176.5 (2)
C7—C6—C1—C2	179.8 (3)	C10—C11—N5—N5	0(12)
C5—C6—C1—N1	−179.3 (3)	O—C8—N1—C1	−179.0 (3)
C7—C6—C1—N1	0.2 (3)	O—C8—N1—C1	−179.0 (3)
C5—C6—C7—N2	−1.8 (5)	C7—C8—N1—C1	−0.1 (3)
C1—C6—C7—N2	178.7 (3)	C2—C1—N1—C8	−179.6 (3)
C5—C6—C7—C8	179.2 (3)	C6—C1—N1—C8	0.0 (3)
C1—C6—C7—C8	−0.2 (3)	N1—C8—O—O	0.0 (2)
O—C8—C7—N2	0.2 (5)	C7—C8—O—O	0.00 (11)

### Hydrogen-bond geometry (Å, °)

**Table:**

*D*—H···*A*	*D*—H	H···*A*	*D*···*A*	*D*—H···*A*
N3—H5···O	0.86	2.10	2.769 (3)	134
N4—H6···N5	0.86	2.61	3.438 (5)	161
N4—H7···O^i^	0.86	2.05	2.906 (4)	173
N1—H4···S^ii^	0.86	2.50	3.350 (3)	169

Symmetry codes:  (i) *x*−1/2, −*y*−1/2, *z*−1/2; (ii) *x*+1/2, −*y*−1/2, *z*+1/2.

## References

[bb1] Brandenburg, K. (2006). *DIAMOND* Crystal Impact GbR, Bonn, Germany.

[bb2] Bruker (2003). *SAINT* Bruker AXS Inc., Madison, Wisconsin, USA.

[bb22] Bruker (2006). *APEX2* Bruker AXS Inc., Madison, Wisconsin, USA.

[bb3] Campaigne, E. & Archer, W. L. (1952). *J. Am. Chem. Soc.* **74**, 5801.

[bb4] Chiyanzu, I., Hansell, E., Gut, J., Rosenthal, P. J., McKerrow, J. H. & Chibale, K. (2003). *Bioorg. Med. Chem. Lett.* **13**, 3527–3530. 10.1016/s0960-894x(03)00756-x 14505663

[bb5] Sheldrick, G. M. (2008). *Acta Cryst.* A**64**, 112–122. 10.1107/S0108767307043930 18156677

[bb6] Westrip, S. P. (2010). *J. Appl. Cryst.* **43**, 920–925.

